# The transcriptional activator Gli2 modulates T-cell receptor signalling through attenuation of AP-1 and NFκB activity

**DOI:** 10.1242/jcs.165803

**Published:** 2015-06-01

**Authors:** Anna L. Furmanski, Alessandro Barbarulo, Anisha Solanki, Ching-In Lau, Hemant Sahni, Jose Ignacio Saldana, Fulvio D'Acquisto, Tessa Crompton

**Affiliations:** 1 Immunobiology Section, Institute of Child Health, University College London, London WC1N 1EH, UK; 2 Centre for Biochemical Pharmacology, William Harvey Research Institute, QMUL, London EC1M 6BQ, UK

**Keywords:** Hedgehog, Gli2, T-cell, TCR, AP-1, NFκB, IL-2

## Abstract

Different tissues contain diverse and dynamic cellular niches, providing distinct signals to tissue-resident or migratory infiltrating immune cells. Hedgehog (Hh) proteins are secreted inter-cellular signalling molecules, which are essential during development and are important in cancer, post-natal tissue homeostasis and repair. Hh signalling mediated by the Hh-responsive transcription factor Gli2 also has multiple roles in T-lymphocyte development and differentiation. Here, we investigate the function of Gli2 in T-cell signalling and activation. Gene transcription driven by the Gli2 transcriptional activator isoform (Gli2A) attenuated T-cell activation and proliferation following T-cell receptor (TCR) stimulation. Expression of Gli2A in T-cells altered gene expression profiles, impaired the TCR-induced Ca^2+^ flux and nuclear expression of NFAT2, suppressed upregulation of molecules essential for activation, and attenuated signalling pathways upstream of the AP-1 and NFκB complexes, leading to reduced activation of these important transcription factors. Inhibition of physiological Hh-dependent transcription increased NFκB activity upon TCR ligation. These data are important for understanding the molecular mechanisms of immunomodulation, particularly in tissues where Hh proteins or other Gli-activating ligands such as TGFβ are upregulated, including during inflammation, tissue damage and repair, and in tumour microenvironments.

## INTRODUCTION

Peripheral T-cell activation, differentiation and clonal expansion are controlled by complex signals, which are initiated by interaction of the T-cell receptor (TCR) with its major histocompatibility complex (MHC)–peptide ligand. For full activation, T-cells require co-stimulation by binding of CD28 to CD80 and CD86 on antigen-presenting cells (APCs). TCR and CD28 ligation lead to a series of TCR-proximal phosphorylation events, the release of intracellular Ca^2+^ stores and triggering of signalling cascades, which activate key transcription factors including the activator protein 1 (AP-1) complex, members of the nuclear factor of activated T-cells (NFAT) family and nuclear factor κB (NFκB) (Huse, 2009). The transcriptional response to TCR signalling controls the subsequent activation and resolution of the adaptive immune response (Thompson et al., 1989; Smith-Garvin et al., 2009; Cheng et al., 2011). Interleukin-2 (IL-2) is a potent growth factor for T-cells *in vitro* and *in vivo* (Smith, 1988; Boyman and Sprent, 2012). Activation of T-cells induces synthesis of IL-2 and upregulation of cell surface CD25 (IL-2Rα), the high-affinity IL-2 receptor subunit, thus providing a feedback loop that enhances IL-2 signalling. Persistent stimulation of T-cells through TCR and IL-2 signalling eventually induces apoptotic pathways, resulting in activation-induced cell death (AICD). The function of TCR, CD28 and cytokine signalling in T-cell activation is well characterised, although the role of other microenvironmental cues in altering local T-cell responses is not well understood. Different secondary lymphoid organs, distinct tissue niches and solid neoplasms each present diverse and dynamic cellular microenvironments, which might provide different signals to local resident or infiltrating T-cells. The influence of non-immune tissue-derived molecules on T-cell activation therefore requires investigation.

Hedgehog (Hh) proteins are secreted inter-cellular signalling molecules that are essential for patterning during fetal development and homeostasis of adult tissues (Neumann, 2005; Ingham and Placzek, 2006; Agathocleous et al., 2007; Crompton et al., 2007; Jiang and Hui, 2008; Le et al., 2008). Hh pathway molecules are expressed in the thymus (Outram et al., 2000; Sacedón et al., 2003), where Hh signalling regulates multiple stages of T-cell development (Outram et al., 2000; Shah et al., 2004; Hager-Theodorides et al., 2005; El Andaloussi et al., 2006; Rowbotham et al., 2007; Rowbotham et al., 2008; Hager-Theodorides et al., 2009; Rowbotham et al., 2009; Drakopoulou et al., 2010; Furmanski et al., 2012; Michel et al., 2013). Gene expression studies have shown that mature splenic T-cells express the Hh signal transduction molecules *Ptch1* and *Smo* (Lowrey et al., 2002; Furmanski et al., 2013). Desert Hh (Dhh) is expressed in spleen (Perry et al., 2009; Lau et al., 2012), and Sonic Hh (Shh) is produced by follicular dendritic cells in spleen and lymph nodes (Sacedón et al., 2005), and by the stroma of several tissues (Sato et al., 1999; Pola et al., 2003; Nielsen et al., 2004; Furmanski et al., 2013). Many tumours secrete Hh ligands, and the pathway is active in wound repair and fibrotic diseases (Jiang and Hui, 2008; Le et al., 2008).

Canonical mammalian Hh signalling is initiated by the binding of Shh, Dhh or Ihh to the cell surface receptor Patched1 (Ptch1) (Marigo et al., 1996). This interaction relieves inhibition of Smoothened (Smo), the Hh signalling transduction molecule (Alcedo et al., 1996), which activates members of the Gli family of transcription factors (Varjosalo and Taipale, 2007). Gli proteins bind to DNA at consensus Gli-family-binding sites and directly modulate target gene transcription. Gli2 is necessary to initiate the Hh signal and acts mainly as a transcriptional activator *in vivo*, but can be processed into truncated isoforms of the full-length Gli2 protein, which activate (Gli2A) or repress (Gli2R) transcription (Aza-Blanc et al., 2000; Bai et al., 2002). Hh ligand binding blocks production of Gli2R, and full-length Gli2 proteins are converted into Gli2A, altering the cellular ratio of Gli2R:Gli2A, which regulates the cellular response to Hh signals. In the absence of ligand, a significant proportion of transcriptionally inactive Gli2 remains in the cytoplasm. Gli1 can function only as a transcriptional activator and is itself a transcriptional target of Gli2A. All Gli proteins contain a nuclear localisation signal (NLS) and nuclear export signal (NES), which control movement between the nucleus and cytoplasm. Upon Hh ligand binding, Gli2A accumulates in the nucleus to activate Hh-dependent transcription (Kim et al., 2009; Hatayama and Aruga, 2012). Interestingly, Gli2 has also been shown to be responsive to TGFβ signal transduction in several cell types (Dennler et al., 2007), and Gli2A function can also be regulated by Akt–PI3K and EGF signalling (Kasper et al., 2006; Riobó et al., 2006).

Here, we show that Gli transcription factors are present in the nucleus and cytoplasm of wild-type (WT) splenic CD4+ cells. To investigate how Gli2-mediated transcription influences TCR signalling in CD4+ T-cells, we used transgenic models where transcription of target genes by Gli2 is either constitutively active (Gli2A) or repressed (Gli2R) (Rowbotham et al., 2007; Rowbotham et al., 2008). The Gli2A and Gli2R transgenes are driven by the *Lck* promoter and so are expressed in T-lineage cells only (Buckland et al., 2000; Shimizu et al., 2001). The transgenes are otherwise identical in sequence and share the zinc finger domains that bind to DNA at consensus Gli-binding sites.

We show that the ability of T-cells to signal, activate, proliferate and respond to IL-2 is impaired in the presence of Gli2A. Our data indicate that Gli2-dependent transcription attenuates T-cell activation by altering the expression of genes important for several key signalling events downstream of TCR ligation. This has implications for our understanding of immune regulation in tissues that express ligands able to activate Gli-dependent transcription.

## RESULTS

### Gli transcription factors are expressed in WT T-cells

**Fig. 1.**
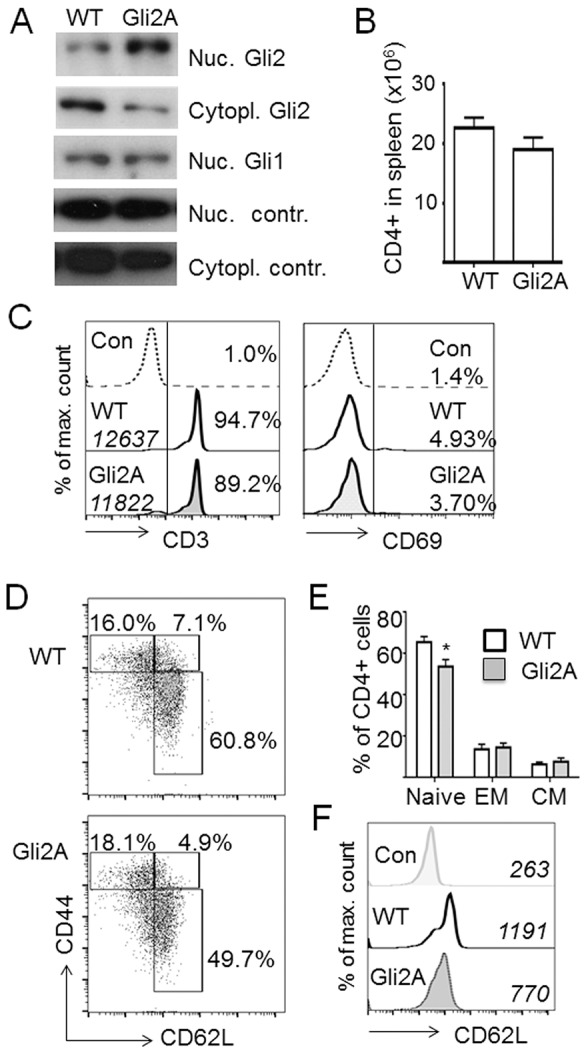
**CD4+ T-cell phenotype of Gli2A mice.** (A) Western blotting with anti-Gli2 or -Gli1 antibody on cell extracts from purified CD4+ splenocytes of WT and Gli2A mice. Nuclear control, anti-laminA antibody; cytoplasmic control, anti-vinculin antibody. (B) Mean±s.e.m. number of CD4+ lymphocytes in spleen from WT (*n*=14) and Gli2A (*n*=15) mice. (C) Histograms showing representative expression of CD3 and CD69 on CD4+ T-cells from spleen of WT and Gli2A mice. The percentage of cells that are CD3+ or CD69+ is also indicated, and the mean fluorescence intensity (MFI) of anti-CD3 staining is shown in italics (*n*=7 pairs, percentage CD3+, *P*<0.03 compared with WT; the MFI of CD3+ cells and percentage of CD69+ is not significantly different from WT). (D) Representative CD62L and CD44 expression profiles on splenic CD4+ cells and (E) percentage (mean±s.e.m.) of CD4+ T-cells from WT (*n*=5) and Gli2A (*n*=5) spleen that are CD62L^hi^ CD44− (naïve, *P=0.03), CD62L+ CD44+ (central memory, CM) and CD62L^−^ CD44+ (effector memory, EM). (F) Histograms show CD62L expression on CD4+ T-cells with MFI in italics (Con, unstained control) from a representative example of *n*=5 pairs. *P*<0.002 for WT compared with Gli2A.

To show that WT T-cells express the essential Hh-responsive transcription factor Gli2, we investigated Gli2 protein expression and localisation by western blotting extracts from CD4+ cells purified from spleen (Fig. 1A). Gli2 was expressed in the cytoplasm and nucleus of *ex vivo* unstimulated WT CD4+ lymphocytes. Nuclear accumulation of Gli2A is associated with active Hh signalling (Kim et al., 2009), therefore we investigated expression of Gli1, a canonical Gli2 target and transcriptional activator. Gli1 protein was also present in WT CD4+ T-cells. The presence of Gli1 protein and nuclear localisation of Gli2 suggest that there is Gli transcriptional activity in WT CD4+ T-cells. These data support the observation that differentiated CD8+ cytotoxic T-cells express Ptch1 and Gli1 protein (de la Roche et al., 2013).

We then examined Gli expression in T-cells from Gli2A transgenic mice (which overexpress the activator form of Gli2 in T-cells). Gli2 and Gli1 were expressed in Gli2A T-cells (Fig. 1A). The ratio of nuclear to cytoplasmic Gli2 was higher in the Gli2A transgenic T-cells compared to WT CD4+ T-cells, consistent with the transgenic expression of a transcriptional activator. Interestingly, we did not observe an increase in nuclear Gli1 protein in the Gli2A cells compared to WT, despite the fact that *Gli1* transcript (and also the *Ptch1* transcript) is upregulated in the transgenic cells (Rowbotham et al., 2007; Furmanski et al., 2013). The Gli transcription factors have distinct tissue-specific functions (Mo et al., 1997; Matise et al., 1998; Bai et al., 2002), and in many tissues it has only been possible to identify a function for Gli1 when *Gli2* is mutated (Park et al., 2000). Our data show that in T-cells, increased Gli2A protein does not lead to a corresponding increase in nuclear Gli1 protein, suggesting that regulatory feedback mechanisms influence protein levels. This would be consistent with the fact that Gli1 deficiency makes little functional impact unless *Gli2* is absent.

Given that WT CD4+ cells express Gli2 and Gli1 directly *ex vivo*, and that Gli2 localised to the nucleus, we next investigated the influence of Gli2 transcriptional activity on CD4+ T-cell phenotype and function.

### Phenotype of T-cells expressing Gli2A

We compared the CD4+ cell phenotype of WT mice and Gli2A transgenic mice. Compared to WT, CD4+ T-cell numbers in Gli2A spleen were not significantly different to those in WT (Fig. 1B), and *ex vivo*, these T-cells expressed similar levels of the TCR-associated signalling molecule CD3 and low levels of the early activation marker CD69 (Fig. 1C). Interestingly, compared to WT, a lower proportion of Gli2A CD4+ cells were CD62L+ CD44− (CD62L is also known as L-selectin) (Fig. 1D), a phenotype associated with naïve T-cells. However, there was no equivalent increase in CD44+ cells (effector memory or central memory) in Gli2A spleen (Fig. 1E), suggesting that Gli2A does not alter the distribution of T-cells among memory subsets. CD44– cells that are also either CD62L^lo^ or CD62L– are seen in the Gli2A CD4+ population. Such cells are not readily detectable in WT spleen. Instead we observed that the presence of Gli2A in CD4+ T-cells downregulated the level of surface CD62L (Fig. 1F).

### Gli2A negatively regulates T-cell activation and proliferation

**Fig. 2.**
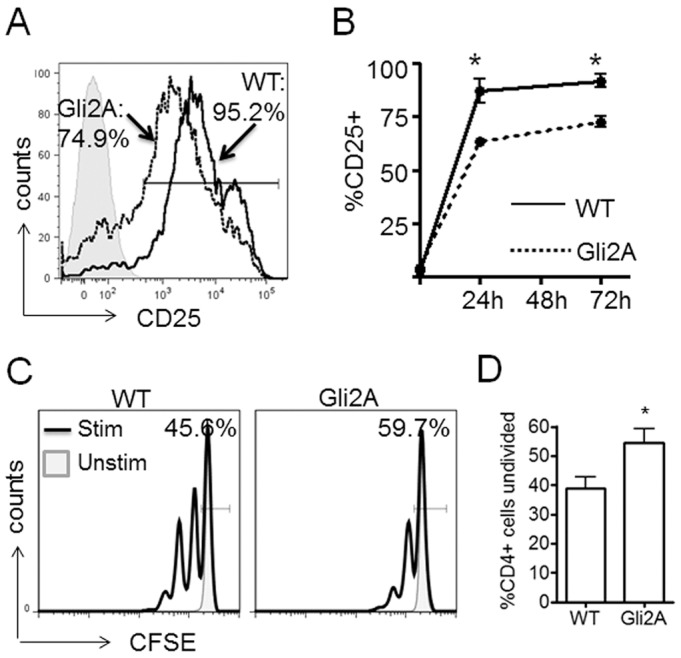
**Gli2-mediated transcription attenuates T-cell activation kinetics.** (A,B) Gli2A and WT splenocytes were cultured with 0.01 μg/ml anti-CD3/CD28 for three days. Cells were stained with anti-CD25 and anti-CD4 antibody. (A) Expression of CD25 on CD4+ cells after 3 days. Histograms show the percentage of CD25+ cells in WT (solid line) and Gli2A (dotted line). (B) Chart shows the percentage (mean±s.e.m.) of CD4+ T-cells that express cell-surface CD25 (*n*=3 mice per group, *P<0.05). (C) CD4+ cell division measured by CFSE dilution at 72 h after activation with anti-CD3/CD28. Histogram bar and label shows the percentage of cells that had not divided. (D) Percentage (mean±s.e.m.) of CD4+ cells that had not divided after 72 h in WT and Gli2A activation cultures, *n*=4, *P=0.04.

We then examined expression of the activation marker CD25 on CD4+ cells treated with antibody against CD3 and CD28 (hereafter anti-CD3/CD28) through a 3-day time-course. In response to stimulation, Gli2A CD4+ cells were less able to upregulate surface CD25 (Fig. 2A,B) than WT cells at any time point examined, suggesting that activation of Gli2A T-cells is defective with slower kinetics throughout the response.

We therefore assessed Gli2A CD4+ cell proliferation following CD3/CD28 ligation. We cultured WT and Gli2A CFSE-labelled splenocytes with anti-CD3/CD28 and analysed dye dilution at 72 h. Consistent with impaired activation, we observed proportionally fewer CFSE^low^ peaks compared to WT cells (Fig. 2C) and an increased percentage of undivided CFSE^hi^ CD4+ cells (Fig. 2C,D) in Gli2A cultures compared to WT. These data complement our previous findings that T-cell-specific repression of Gli-dependent transcription increases TCR-driven T-cell activation and proliferation (Rowbotham et al., 2008).

### Gli-dependent transcription alters cell survival

**Fig. 3.**
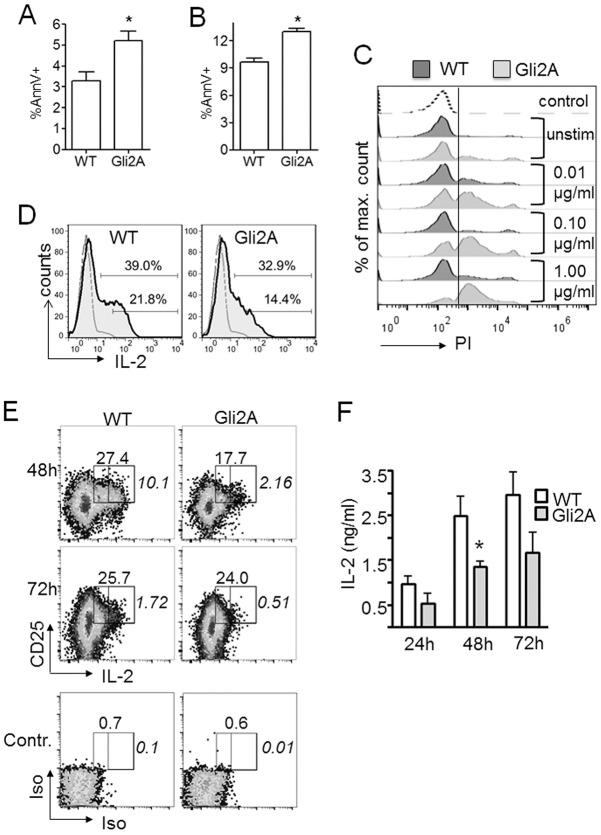
**Gli2A-driven transcription enhances apoptosis and impairs IL-2-responsiveness during division.** (A) Percentage (mean±s.e.m.) of CD4+ AnnV+ (early apoptotic cells) as determined by live gating from splenocyte activation cultures (24 h, *n*=5 experiments, *P=0.02) and (B) percentage of CD4+ AnnV+ cells in unstimulated cultures (72 h, *n*=3, *P=0.02). (C) Propidium iodide (PI) staining of CD4+ splenocytes at 24 h after stimulation with anti-CD3/CD28 (0, 0.01, 0.1 or 1.0 µg/ml; 0, unstimulated). Histograms are representative of two experiments. Dotted, background staining; dark-grey, WT; light-grey, Gli2A. (D) Intracellular IL-2 staining in fresh CD4+ splenocytes following 3 h activation with PMA (50 ng/ml), ionomycin (500 ng/ml) and Brefeldin A (2 ng/ml). Bars on shaded histograms show the percentage of IL-2+ and IL-2^hi^ cells, gated on CD4+ cells; the dashed grey line shows background staining. A representative example from five experiments is shown (IL-2hi, WT, 25.0%±0.6, Gli2A: 19.9±1.5, *P*=0.009). (E) Purified CD4+ cells were cultured with anti-CD3/CD28-coated beads for up to 3 days. Surface CD25 and intracellular IL-2 were measured by flow cytometry at the times indicated, the percentage of CD25+ IL-2+ cells (large gate) and CD25+ IL-2^hi^ cells (smaller gate, labelled in italic numerals). Gates were set according to isotype control staining of CD4+ cells (lower panels). Results are representative of three experiments (*n*=3, WT versus Gli2A percentage for CD25+ IL-2+ cells at the 48 h time-point, *P*<0.01 compared with WT). (F) ELISA showing mean±s.e.m. IL-2 produced by purified CD4+ cells cultured with anti-CD3/CD28-coated beads (WT, *n*=5; Gli2A, *n*=4). *P=0.04.

Our data show that Gli-dependent transcription influences T-cell activation and proliferation (Fig. 2; Rowbotham et al., 2007; Rowbotham et al., 2008). Population expansion is a dynamic process and the overall outcome will depend on the balance between activation, division and cell death. We therefore investigated apoptosis in Gli2A activation cultures by assessing cell surface binding of Annexin V (AnnV), a marker of membrane-flipping during early apoptosis. A higher percentage of Gli2A CD4+ cells bound AnnV relative to WT after 24 h in activation cultures, indicating an increased proportion of cells undergoing the early stages of apoptosis (Fig. 3A). In order to determine whether this applies only to death of activated T-cells, we stained control, unstimulated splenocyte cultures at 72 h with AnnV. Again, Gli2A CD4+ cell populations displayed greater AnnV staining than WT, indicating increased apoptosis (Fig. 3B). To further investigate, we measured propidium iodide staining in WT and Gli2A CD4+ lymphocytes after 24 h in culture with increasing anti-CD3/CD28 dose (Fig. 3C). Propidium iodide is actively excluded from live cells; hence, propidium iodide uptake represents membrane permeability and late-stage apoptosis. As with AnnV, we noted an increased proportion of cells undergoing apoptosis in unstimulated and stimulated (0.01 µg/ml) Gli2A cultures compared to WT. Interestingly, as anti-CD3/CD28 dose increased log-fold we observed large increases in propidium iodide staining in Gli2A CD4+ cells indicating widespread apoptosis in the cultures, whereas the majority of WT cells were still able to exclude propidium iodide.

Taken together, these data suggest that Gli-dependent transcription in T-cells impairs cell survival and increases the likelihood of cell death.

### Gli2 activity impairs IL-2 production by activated T-cells

T-cell activation stimulates the production of IL-2, a potent T-cell growth and survival factor. As Gli2A cells display attenuated activation, a proliferation defect and an increased tendency to undergo apoptosis, we tested the capability of Gli2A cells to produce IL-2. After a short mitogenic stimulation of splenocytes directly *ex vivo*, we found that significantly fewer CD4+ Gli2A cells expressed intracellular IL-2 compared to WT (Fig. 3D). To confirm that this effect was cell intrinsic and not due to bystander effects from other splenocytes, we purified CD4+ T-cells and cultured them with anti-CD3/CD28-coated beads for 72 h. We found that compared to WT, a lower proportion of Gli2A cells expressed intracellular IL-2 at 48 h (Fig. 3E). Strikingly, IL-2^hi^ CD25+ cells, which are likely to be fully activated and signalling in an autocrine/paracrine manner, were significantly diminished in Gli2A cultures compared to WT (Fig. 3E) at both 48 h and 72 h. Gli2A culture supernatants contained less IL-2 protein than WT cultures as measured by ELISA at 24 h, 48 h and 72 h. This was most pronounced and statistically significant at 48 h (Fig. 3F).

### Exogenous IL-2 does not rescue the phenotype of Gli2A cells

**Fig. 4.**
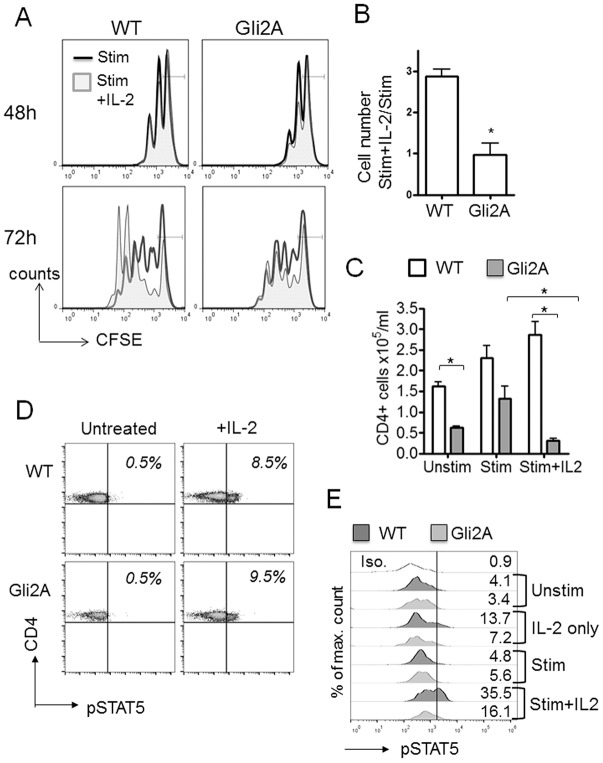
**Gli2-dependent transcription decreases IL-2 production and CD25 (IL2R) upregulation.** (A) Histograms representing dye dilution measured by flow cytometry over time in CFSE-loaded CD4+ splenocytes activated with 0.01 μg/ml anti-CD3/CD28 without (stim, black line) or with 10 U/ml rIL-2 (stim+IL-2, shaded). Histograms show overlays of stimulated and stimulated +IL-2 cultures, bar markers show the peak representing the undivided population. (B) Flow Jo software was used to calculate cell proliferation in cultures. The number of cells in each generation (peak) was estimated and the relative effect of IL-2 on cultures was calculated. The bar chart shows number of cells undergoing more than three divisions in stim+IL-2 cultures relative to stim alone (mean±s.e.m. *n*=4 pairs, *P=0.001). (C) Number of live CD4+ cells remaining in culture at 72 h (*n*=3, *P<0.03) and (D) phospho-STAT5 staining in CD4+ splenocytes that were unstimulated (left panels) or following 15 min culture with 10 ng/ml IL-2 (right panels; WT *n*=3, 8.9±1.5%; Gli2A *n*=4, 8.4±1.6%, which is not significantly different from WT). (E) Phospho-STAT5 staining in CD4+ splenocytes cultured for 48 h with IL-2 or anti-CD3/CD28+IL-2. Histograms showing the percentage of phospho-STAT5+ cells, representative of two independent experiments. WT, dark-grey; Gli2A, light grey.

Activated Gli2A cells produce less IL-2 than WT cells, and show decreased proliferation and increased apoptosis. To test whether this phenotype can be rescued by exogenous IL-2, we performed proliferation experiments where cells were cultured with anti-CD3/CD28 in the presence or absence of recombinant-IL-2 (rIL-2). Addition of rIL-2 did not affect the proportion of dividing cells in WT or Gli2A cultures after 48 h (Fig. 4A). By 72 h the presence of rIL-2 had significantly enhanced the proliferation and/or survival of WT cells (Fig. 4A). However, we observed no significant enhancement in proliferation of Gli2A cells when exogenous IL-2 was added. We calculated the relative number of cells having divided more than twice by 72 h, and compared this between samples cultured with and without exogenous IL-2. In WT cultures, we found that approximately threefold more cells had undergone multiple divisions when IL-2 was added together with anti-CD3/CD28, compared to anti-CD3/CD28 alone. However, this was not observed in Gli2A cultures (Fig. 4B).

To assess the impact of rIL-2 on maintenance of our cultures, we assessed cell viability and number. At 72 h, all Gli2A cultures displayed lower numbers of CD4+ cells compared to WT (Fig. 4C). The highest cell numbers were observed in WT cultures treated with anti-CD3/CD28 and rIL-2. However, there was a marked decrease in CD4+ cell number in the equivalent Gli2A cultures (Fig. 4C).

These data indicate that IL-2 responsiveness is attenuated when Gli2-dependent transcription is active. Gli2A cells proliferate less well than WT cells (Fig. 2) and produce less IL-2 upon TCR stimulation (Fig. 3), and their proliferation defect cannot be rectified by the addition of exogenous IL-2. In contrast to WT cells, Gli2A cells do not fully upregulate CD25 upon activation (Fig. 2A,B). CD25 is an important subunit of the high-affinity receptor for IL-2. Therefore, because CD25 upregulation is lower on Gli2A than on WT cells, it seems likely that Gli2A cells express a lower density of high-affinity IL-2R on the cell surface than WT. IL-2, either added to or produced by Gli2A cells in culture might therefore be saturating due to decreased IL-2R availability. Alternatively, Gli2A cells might have defective intracellular IL-2–IL-2R signalling.

### Gli2 activity does not affect the ability of CD4+ cells to transduce IL-2 signals through STAT5

To determine whether Gli2A cells are less able to transduce IL-2 signals independently of TCR signalling and subsequent CD25 upregulation (i.e. in the presence of IL-2 alone, without any other T-cell stimulation), we cultured fresh splenocytes directly *ex vivo* for 15 min with recombinant IL-2 and measured expression of intracellular phosphorylated signal transduction and activator of transcription 5 (pSTAT5), a major transcription factor that is activated downstream of IL-2 signalling (Gilmour et al., 1995). We found no significant difference in the proportion of cells expressing pSTAT5 between WT and Gli2A CD4+ cells (Fig. 4D), suggesting that Gli2A cells do not have an inherent inability to transduce IL-2 signals that result in phosphorylation of STAT5. To assess the impact of T-cell activation on STAT5 phosphorylation in WT and Gli2A cells, we measured pSTAT5 levels in CD4+ cells from activation cultures (Fig. 4E). As expected, unstimulated and anti-CD3/CD28-stimulated CD4+ cells showed similar pSTAT5 expression in WT and Gli2A cultures. However, we noted a marked decrease in pSTAT5 expression in Gli2A cells from cultures stimulated with anti-CD3/CD28 in the presence of IL-2. These experiments indicate that the inability of Gli2A T-cells to upregulate CD25 and proliferate efficiently on CD3/CD28 ligation is not simply the result of reduced IL-2 signalling, but is rather the direct influence of Gli-mediated transcription on TCR–CD28 driven cell activation. We, therefore, investigated TCR signalling in this model.

### Gli2-driven transcription regulates the T-cell Ca^2+^ flux

**Fig. 5.**
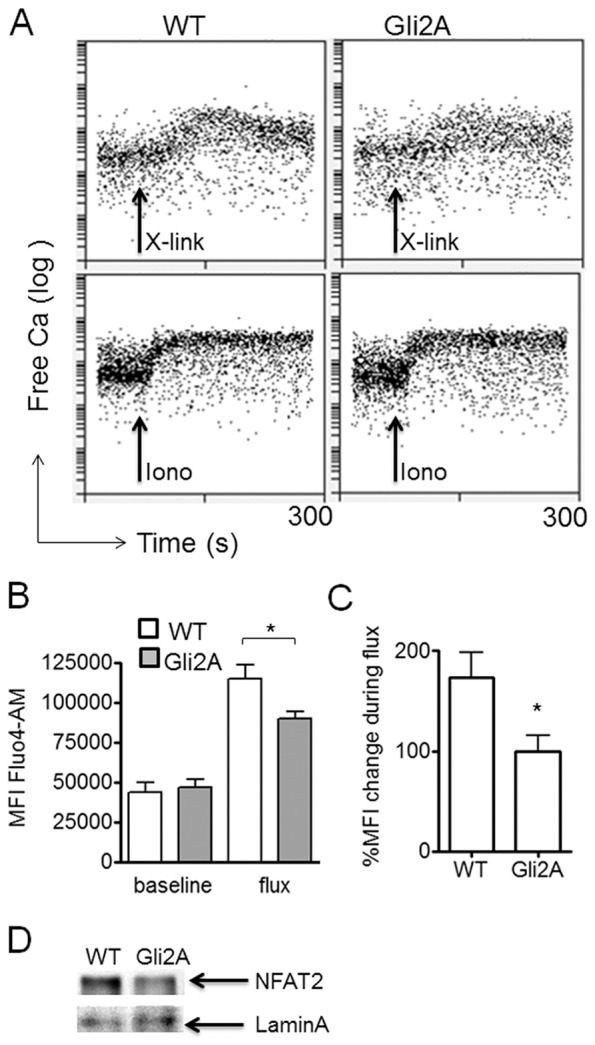
**Gli-mediated transcription regulates the T-cell Ca^2+^ flux.** (A) Splenocytes were loaded with Fluo-4-AM (3 µM) and surface stained. Cells were incubated with hamster anti-mouse-CD3 antibody (3 µg per 10^6^ cells) for 20 min on ice, washed, resuspended in PBS containing Ca^2+^ and incubated at 37°C prior to acquisition by flow cytometry. Cells were stimulated with 9 µg cross-linking anti-hamster-IgG antibody or 0.1 µg/ml ionomycin. Fluorescence intensity in FL-1 (Fluo-4-AM) was recorded during the 60 s baseline and for a further 4 min through the peak of flux (B, *n*=5, *P=0.03). The relative change in MFI [100×(peak−baseline)/baseline] was calculated (percentage flux) for (C) WT and Gli2A (mean±s.e.m., *P=0.02). (D) Western blotting of NFAT2 in nuclear extracts from purified CD4+ cells, which were activated for 24 h with 2.5 µg/ml plate-bound anti-CD3/CD28. Loading control, anti-laminA antibody.

Upon TCR ligation, T-cells undergo a sustained influx of Ca^2+^ ions across the plasma membrane, which stimulates the release of Ca^2+^ from intracellular stores, triggering signalling pathways and the activation of transcription factors including NFκB and NFAT. Impaired Ca^2+^ flux has been shown to diminish events downstream of TCR activation necessary for proliferation and survival, including IL-2 production (Feske, 2007). In order to determine whether Gli-dependent transcription influences the Ca^2+^ flux, we activated Gli2A T-cells and monitored Ca^2+^ entry into cells loaded with Fluo-4-AM, a Ca^2+^-binding fluorescent dye. Baseline fluorescence was measured for 60 s prior to TCR stimulation. We observed a sustained Ca^2+^ flux in WT T-cells following cross-linking of surface CD3 molecules (Fig. 5A, upper panels). In comparison, Gli2A cells showed a diminished flux of lower magnitude (Fig. 5A–C). Gli2A cells were able to undergo a Ca^2+^ flux that was comparable to WT cells upon stimulation with ionomycin, a Ca^2+^ ionophore (Fig. 5A, lower panels), indicating that the impaired fluxes seen with anti-CD3 activation were due to defective TCR signalling.

NFAT transcription factors are crucial regulators of T-cell function during activation. TCR-induced Ca^2+^ flux triggers dephosphorylation and translocation of NFAT proteins to the nucleus, where they modulate T-cell transcription. In order to assess whether the impaired Ca^2+^ flux in Gli2A cells affected nuclear expression of NFAT, we performed western blotting for NFAT2 on nuclear extracts of WT and Gli2A CD4+ cells following anti-CD3/CD28 stimulation (Fig. 5D). As expected given their weak Ca^2+^ flux, Gli2A cells showed lower expression of nuclear NFAT2 after stimulation.

Our data show that Gli2, a transcription factor that is expressed in WT cells, modulates T-cell biology through mechanisms downstream of the TCR. We therefore sought to examine the transcriptional mechanisms involved in the influence of Gli-dependent transcription on TCR signalling during the early stages of T-cell activation.

### Expression of Gli proteins in Gli2R CD4+ T-cells

**Fig. 6.**
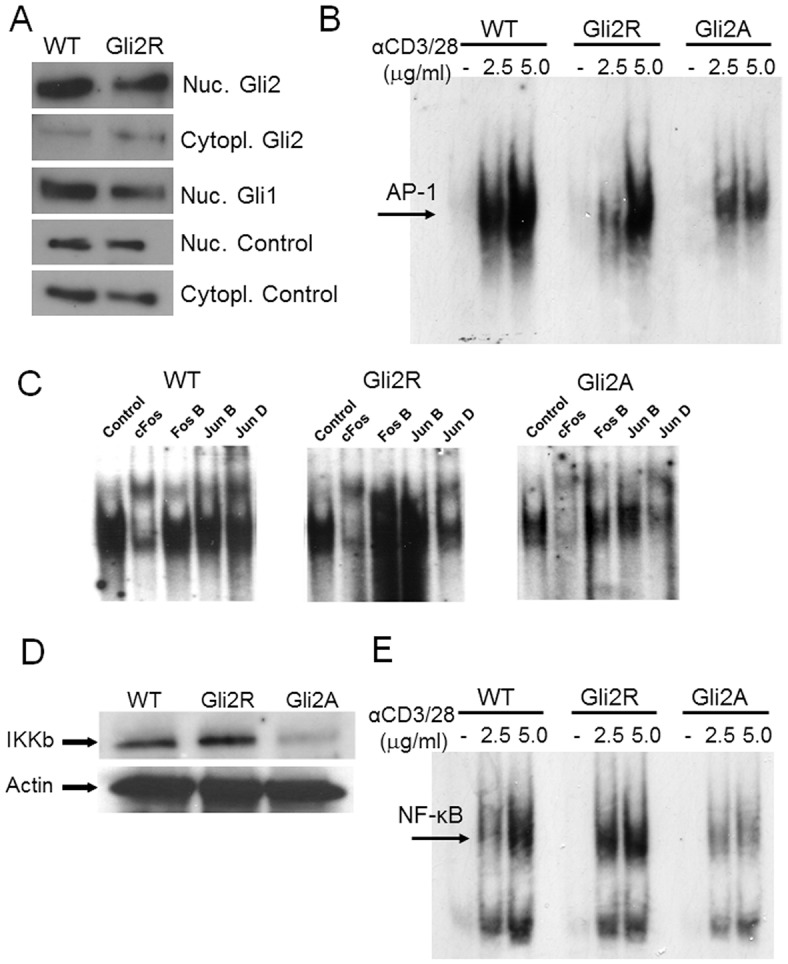
**Gli2A-driven transcription attenuates expression and DNA-binding activity of key T-cell transcription factors.** (A) Western blotting as in Fig. 1A on extracts from purified CD4+ cells from WT and Gli2R mice. (B) AP-1 activity (arrow) was assessed by EMSA in lymphocytes from WT (*n*=3), Gli2R (*n*=3) and Gli2A (*n*=3) mice activated with 1.25, 2.5 and 5 µg/ml plate-bound anti-CD3/CD28. (C) Supershift assays were performed with anti-Fos and anti-Jun antibodies following EMSA. (D) Protein expression of the IKKβ subunit was assessed by western blotting of CD4+ cell lysates from WT (*n*=3), Gli2R (*n*=3) and Gli2A (*n*=3), anti-actin blotting was performed as the loading control. (E) NFκB activity was probed by EMSA as described above, where cells were activated with 2.5 µg/ml or 5 µg/ml anti-CD3/CD28 as indicated.

We have shown that Gli2A cells highly express Gli2 protein in T-cell nuclear extracts (Fig. 1A). These cells also constitutively express high levels of *Ptch1* transcript, a Hh-target gene (Furmanski et al., 2013). Additionally, we have shown that Gli2R transgenic CD4+ cells are refractory to upregulation of *Ptch1* on Shh treatment (Rowbotham et al., 2008), and therefore that physiological Hh signalling is inhibited in these cells. In contrast to Gli2A cells, T-cells from Gli2R transgenic mice show an enhanced activation phenotype compared to WT controls, and displayed increased proliferation after 72 h in culture with anti-CD3/CD28 (Rowbotham et al., 2008). Gli2A and Gli2R proteins are isoforms of the same transcription factor but might reside in different cellular locations due to their distinct functions. We therefore investigated Gli2 protein expression in Gli2R CD4+ cells by western blotting of cell extracts. As expected, Gli2R cells expressed Gli2 (Fig. 6A). Interestingly, there was a lower nuclear to cytoplasmic ratio of Gli2 compared to WT cells, and Gli2R cells also displayed lower abundance of Gli1 protein, itself a canonical Gli-target (Fig. 6A), reflecting the repressor function of the transcription factor.

### Regulation of CD4+ T-cell transcription by Gli2

We have shown that Gli proteins are expressed in WT T-cells, and that the Gli2A and Gli2R transgenes modulate T-cell transcription and function. We therefore investigated which genes are regulated by Gli2 in lymphocytes during TCR stimulation. We measured Gli2-dependent transcription in fresh and anti-CD3/CD28-activated CD4+ T-cells from spleen of WT, Gli2A and Gli2R mice by microarray (GSE33156, GEO depository) as described previously (Furmanski et al., 2013). We analysed the resting and TCR-stimulated T-cell data sets separately in order to identify genes regulated by Gli2 in both the absence and presence of a TCR signal. Gene lists were obtained from normalised fold-change analyses or by moderated *t*-statistics (eBayes Limma analysis) with false discovery rate and adjusted *P*<0.05.

### Identification of new Gli target genes in unstimulated T-cells

Both transgenic strains carry a transgene encoding *Gli2* and share consensus DNA-binding sequences in target genes. We aimed to identify potential direct Gli target genes in CD4+ T-cells directly *ex vivo*. We therefore filtered gene lists by screening genes with an expression pattern across genotypes correlating to *Ptch1*, a canonical Gli target gene in T-cells (Furmanski et al., 2013), to identify 33 genes upregulated in Gli2A and downregulated in Gli2R unstimulated T-cells (supplementary material Table S1). We used the DAVID v6.7 bioinformatics resource (Huang et al., 2009) to cluster these genes into seven functional groupings according to gene annotations. Over a third of these genes encoded membrane-bound proteins and receptors, several of which are involved in the regulation of growth, differentiation or cell death (*Igf1r*, *Ifitm3*, *C6*, *Mmp13*, *Ptlp* and *Qk*) and transport (*Slc1a3*, *Slc25a39*, *Slc16a9*). Others functional groupings included signal transduction, catabolic processes, proteolysis and ion or nucleotide binding. Several genes positively regulated by Gli2A have been implicated in T-cell function: *Rsad2* (also known as *viperin*) is an anti-viral protein and facilitator of Th2 cytokine production. *Ifitm3*, *Hfe* and *Tnfsf12* (also known as *TWEAK*) were also positively regulated by Gli-dependent transcription.

We identified six genes downregulated in Gli2A and upregulated in Gli2R unstimulated CD4+ cells (supplementary material Table S1). *Hspa1a* and *Hspa1b* are anti-apoptotic and involved in NFκB activation. Interestingly, *Igfbp4* and *Sostdc1* both act as BMP or Wnt pathway inhibitors, demonstrating the complex relationship between these major morphogenic pathways. These genes are unlikely to be direct targets of Gli2, as expression decreases when the transcriptional activator form of Gli2 is present, but might be targets of currently unidentified intermediate repressor genes, which are themselves targets of Gli2.

### Gli-mediated transcription in resting and activated T-cells controls expression of morphogenic, developmental and immune cell signalling genes

Array data were further analysed using a cut-off of a 1.5-fold difference in gene expression between genotypes, for unstimulated and stimulated datasets (supplementary material Fig. S1). This showed that several hundred genes were regulated by Gli2. Anti-CD3/CD28 stimulation reduced the number of transcripts that were differentially expressed between genotypes, most likely due to the potent effect of TCR signalling on the T-cell transcriptome. The Gli2A and Gli2R transgenes might not control expression of an identical set of genes in a reciprocal manner, as the cellular location, transcription-complex-binding partners, stability and secondary indirect effects of the Gli2A and Gli2R transcription factor isoforms might differ. To widen our analysis, we identified genes that were differentially expressed in either transgenic strain compared to all other samples. Unstimulated Gli2A T-cells showed upregulation of genes encoding important signalling molecules, including *Tgfb3*, *Sox4*, *Dusp16* and *Hoxa1.* Members of related morphogen signalling pathways, including *Bmpr2* and *Wnt5b*, were also upregulated when Gli2-mediated transcription was active. Gli2A expression in T-cells caused downregulation of genes encoding heat-shock proteins (*Hsp90aa1*, *Hspa1a* and *Hspa1b*), and most interestingly, molecules important in T-cell signalling including *Jun*, *Fos*, *Fosb*, *Lef1*, *Ikbkb* and *Rasgrp1* (supplementary material Fig. S1, selected genes annotated only for clarity). *Sell*, encoding CD62L was also downregulated in Gli2A cells. This reflects our observations that Gli2A T-cells express lower levels of cell-surface CD62L (Fig. 1D,F) and suggests that the CD62L and CD44 phenotype of Gli2A CD4+ cells is influenced by Gli2 at a transcriptional level, rather than by shedding of CD62L protein (Kahn et al., 1994; Hafezi-Moghadam et al., 2001). These genes are all likely to be targets of intermediate repressor genes, which are themselves targets of Gli2. The expression of Gli2R in T-cells caused downregulation of morphogenic genes including *Sox6*, *Fzd7*, *Hoxd1* and *Nkx1.2*. Other genes repressed by Gli2R included several with important roles in the regulation of immune cell signalling (*Il1b*, *Cd24a*, *Vsig4* and *Bcl11a*).

When T-cells were stimulated with anti-CD3/CD28 for 6 h, we observed that several genes upregulated in the Gli2A or downregulated in the Gli2R groups encoded molecules involved in immune cell signalling and apoptosis, including *Csf2*, *Cd24a*, *Fasl* and *Jdp2*. As in unstimulated Gli2A cells, regulators of morphogenesis, growth and/or differentiation (*Igf1r*, *Tgfb3*, *Hoxa1*, *Bmpr1b* and *Sox4*) were upregulated in stimulated Gli2A cells, and *Igfbp4* and *Jun* were downregulated. Interestingly, *Il6*, *Tgfbi* and *Il17a* were repressed by the presence of Gli2R (supplementary material Fig. S1, selected genes only are labelled for clarity), suggesting that Gli2-dependent transcription might play a role in Th17 cell differentiation.

*Il2ra* and *Il2* expression was similar in stimulated Gli2A and WT cells, confirming that the influence of Gli2A on activation-induced upregulation of cell surface CD25 and intracellular IL-2 protein (Figs 2–4) is not a direct transcriptional consequence of the transgene, but rather the consequence of the overall reduction in T-cell activation. This indicates that the failure of Gli2A cells to fully upregulate CD25 and synthesise IL-2 (Figs 2–4) is not an artefact due to constitutive overexpression of Gli2, as expression of these genes is not decreased by the presence of Gli2A in T-cells.

We confirmed relative gene expression by quantitative real-time PCR (qPCR) using RNA left over from array experiments (not shown) and from additional samples in independent experiments to validate our microarray results. *Tgfb3*, a gene identified in the microarray experiment as upregulated in Gli2A cells and downregulated in Gli2R cells compared to WT was found to follow this expression pattern in additional samples (supplementary material Fig. S2A). This transcript encodes TGFβ3, a TGFβ family member, expressed by CD4+ T-cells and important in Th17 cell pathogenesis (Lee et al., 2012). There is cross-talk between TGFβ and Shh–Gli signalling pathways; TGFβ can activate GLI2 expression in several cell types (Dennler et al., 2007), and *GLI2* directly binds and activates transcription of the *TGFB1* gene in human CD4+ T-cells (Furler and Uittenbogaart, 2012). Our data strongly suggest that *Tgfb3*, among other TGF-related genes, is a direct transcriptional target of Gli2 in murine CD4+ cells, uncovering further overlap of these pathways.

*Rasgrp1* showed the opposite expression pattern; it was downregulated in Gli2A and upregulated in Gli2R cells. This indicates that its expression is negatively regulated by Gli2-mediated transcription (supplementary material Fig. S2B), most likely through an intermediate repressor gene. Other potential Gli-target genes (supplementary material Fig. S2C; *Igf1r*, *Hoxa1*, *Ifitm3*) or targets of intermediate repressors (supplementary material Fig. S2D; *Igfbp4*, *Ikbkb*, *Hspa1a*, *Nfkbiz*) were also confirmed by qPCR.

### Major components of the TCR signalling pathway are differentially expressed when Gli2 is active or repressed

*Rasgrp1* encodes an important nucleotide exchange factor that specifically activates *Ras*, a major upstream component of the ERK (MAPK) pathway. Rasgrp1 controls T-cell development, differentiation and activation (Reynolds et al., 2004; Priatel et al., 2006; Priatel et al., 2010). We have previously shown defective ERK activation in Gli2A T-cells (Rowbotham et al., 2007). Decreased expression of *Rasgrp1* (supplementary material Fig. S2B) could be one mechanism by which Gli2A negatively regulates T-cell activation. Downstream of the TCR, Ras–ERK–MAPK signalling culminates in the activation of key T-cell transcription factors including AP-1. We therefore examined the activity of AP-1 by performing an electrophoretic mobility shift assay (EMSA) following activation of lymphocytes with anti-CD3/CD28. Interestingly, we found decreased binding of AP-1 probes to Gli2A lymphocyte nuclear extracts compared to WT, indicating impaired DNA-binding activity of AP-1 in T-cells from these mice (Fig. 6B). AP-1 is made up of complexes of Fos and Jun proteins. We therefore performed gel supershift assays to assess the composition of bound AP-1 in WT, Gli2A and Gli2R cells by blotting with antibodies against cFos, Fos B, Jun B and Jun D. In all cases, Gli2A cells showed decreased expression of these proteins on TCR–CD28 ligation (Fig. 6C), consistent with the overall reduction of AP-1 activation (Fig. 6B). In contrast, although overall Gli2R transgenic cells showed somewhat decreased AP-1 activity compared to WT (Fig. 6B), the specific contribution of FosB and JunB to AP-1 was increased. Thus, Gli2 activity regulates the expression of these major transcription factors in T-cells.

*Ikbkb* encodes IKKβ, a protein forming part of the IκB kinase (IKK) complex, which phosphorylates inhibitors of NFκB, allowing translocation of the active NFκB transcriptional complex to the nucleus. Expression of *Ikbkb* transcript was negatively regulated by Gli2-mediated transcription (supplementary material Fig. S2D). We used western blotting to examine expression of IKKβ protein in WT, Gli2A and Gli2R CD4+ cell lysates. There was increased expression of IKKβ in Gli2R samples, whereas Gli2A showed lower levels than the WT sample (Fig. 6D). We therefore examined activity of NFκB in nuclear extracts by EMSA. Binding was severely diminished in the Gli2A sample compared to WT, whereas Gli2R extracts showed enhanced binding compared to WT, particularly at the lower concentration of stimulating anti-CD3/CD28 antibody (Fig. 6E).

We therefore show that Gli2A activity in T-cells impaired full T-cell activation by at least three different mechanisms: (1) diminished Ca^2+^ flux and decreased nuclear NFAT2, (2) decreased AP-1 activation and (3) impaired activation of NFκB.

## DISCUSSION

Here, we showed that Gli2A-mediated transcription in T-cells attenuated T-cell signalling and T-cell activation on CD3/CD28 ligation. We investigated the mechanism of this effect, and showed that increased Gli2 activation caused a reduction in AP-1 activation and NFκB activation downstream of TCR signalling. In contrast, inhibition of physiological Gli2-mediated transcription, in Gli2R transgenic cells, led to an increase in NFκB activation, and a change in the molecular composition of AP-1. Impairment of the activity of important signalling pathways such as Ras–ERK–AP-1 and IKK–NFκB by Gli2A would influence T-cell biology, potentially affecting immune cell differentiation and adaptive immunity. Subsequent dysregulation of IL-2 receptor upregulation, signalling and/or secretion upon weakened TCR stimulation is likely to further limit T-cell responses, providing one explanation for the enhanced apoptosis and decreased number of Gli2A T-cells surviving in culture.

Gli2A cells display normal basal levels of intracellular Ca^2+^; however, they lack the ability to undergo a full flux compared to WT cells upon TCR stimulation, and show decreased nuclear NFAT2 (Fig. 5). This might point to defective intracellular Ca^2+^ stores or ion channel pumps and is something that requires further investigation. The alteration of AP-1 and NFκB activity and impairment of Ca^2+^ fluxing by Gli-dependent transcription are important findings, contributing to our understanding of how Hh and/or Gli signals act to ‘tune down’ TCR signalling, as observed previously during T-cell development and activation (Outram et al., 2000; Rowbotham et al., 2007; Rowbotham et al., 2008; Rowbotham et al., 2009; Drakopoulou et al., 2010; Furmanski et al., 2012).

We have previously reported that Gli2A transcriptional activity in T-cells directly increases the production of IL-4 and subsequent expression of Gata3, promoting differentiation to the Th2 lineage (Furmanski et al., 2013). It is interesting to note that differentiation of naïve CD4+ T-cells to Th2 cells is associated with lower TCR signal strength. TCR signals triggered by low cognate peptide concentration stimulate weak and transient ERK activation, allowing Gata3 and IL-4 expression, which favours Th2 differentiation (Yamane et al., 2005). Therefore, another mechanism for Gli2A to promote Th2 differentiation is by decreasing the strength of the TCR signal by attenuating ERK activation.

Microarray profiling revealed additional wide-ranging effects of Gli2-driven transcription on T-cell gene expression, including positive regulation of several genes involved in T-cell function. These include *Tgfb3*, which is important in Th17-mediated autoimmunity (Lee et al., 2012), *Igf1r*, which is highly expressed in T-ALL cells (Medyouf et al., 2011), *Rsad2* (*viperin*), which is a regulator of viral infection (Seo et al., 2011), *Cd24a*, which is important in T-cell homeostasis (Li et al., 2004) and *Dusp16*, which is a negative regulator of JNK in T-cells (Kumabe et al., 2010). It therefore seems likely that Hh and/or Gli signalling has uncharacterised roles in the modulation of autoimmunity and response to infection.

Hh- and Gli-related molecules or genes involved in development and oncogenesis in other tissues were also found to be differentially regulated in Gli2A and Gli2R T-cells including *Ptch1*, *Bmpr2*, *Wnt5b*, *Hoxa1* and *Sox4*. Our data and others (Lowrey et al., 2002) indicate a role for Hh–Gli signalling in modulating cell division and apoptosis. It is therefore timely and important to examine the role of Hh signalling and/or Gli-mediated transcription in T-cell malignancies, particularly as subtypes of lymphocytic leukaemia have been shown to have differing Hh dependency (Decker et al., 2012).

The role of Hh signalling in influencing the outcome of peripheral T-cell activation in both human and mouse is controversial. In some studies, addition of recombinant Shh to T-cell cultures activated with sub-optimal concentrations of anti-CD3/CD28 led to increased activation and proliferation, whereas anti-Hh antibody reduced cell proliferation (Lowrey et al., 2002; Chan et al., 2006). However, conditional deletion of Smo or Ptch1 had no significant effect on T-cell proliferation kinetics when anti-CD3 stimulating antibodies were used *in vitro* (El Andaloussi et al., 2006; Michel et al., 2013). Our data shows that Gli2-mediated transcription attenuates T-cell activation and proliferation. Our current and previous studies (Rowbotham et al., 2007; Rowbotham et al., 2008) largely use a T-cell-specific transgenic approach, in order to rule out cell-extrinsic secondary mechanisms and non-specific effects from high concentrations of recombinant protein, such as those from suspected endotoxin contamination of commercially available recombinant Shh (Wakelin et al., 2008). Additionally, there is emerging evidence in some cell types for non-canonical Hh–Gli signalling, such as Smo-independent signalling, Hh-dependent Smo-dependent Gli-independent signalling, or Hh-independent activation of Gli transcription (Hui and Angers, 2011; Brennan et al., 2012). Our Gli2A and Gli2R transgenic systems are designed to investigate the specific influence of Gli-mediated transcription on T-cell gene expression and function. This will reflect Hh-dependent transcription, as Gli-mediated transcriptional activation is downstream of canonical Hh signalling. Indeed, we have previously shown that canonical Hh target genes are up- and down-regulated in Gli2A and Gli2R cells respectively, and that in T-cells, *Smo* mimics this expression pattern (Furmanski et al., 2013). However, as T-cells lack cilia (Finetti et al., 2011), there are likely to be differences in Hh signal transduction in T-cells compared to ciliated cells. In ciliated cells, upon ligand binding to Ptch1, the signal transduction molecule Smo is trafficked along the primary cilium, and movement of Smo is believed to be essential for signalling (Rohatgi et al., 2007). In the case of T-cells, the absence of cilia could explain apparent inconsistencies between conditional Smo^−/−^ (El Andaloussi et al., 2006), conditional Ptch1^−/−^ (Michel et al., 2013) and Gli2A or Gli2R transgenic models. Furthermore, the potential for activation of Gli proteins by non-Hh signalling pathways, such as through TGFβ signalling, could explain discrepancies in observations made in Gli2A and Gli2R systems and those using recombinant Hh protein in cell culture. These questions require further investigation.

The specific activity of morphogens like Hh family proteins varies with cellular context. The complex role for Hh–Gli signalling in T-cell activation requires further dissection, and might have wide-ranging effects on the immune response in health and disease. Increased expression of Hh protein or other molecules that can trigger Gli activity during tissue renewal could attenuate local T-cell responses, potentially aiding repair or skewing local immune responses. We have previously shown that Hh proteins are upregulated in the lung following the induction of allergic lung disease (Furmanski et al., 2013), and Shh is known to be upregulated in chronic pulmonary fibrosis (Stewart et al., 2003), in inflammatory gut disease (Nielsen et al., 2004), in wound healing (Le et al., 2008) and after tissue ischemia (Pola et al., 2003). Importantly, many cancers overexpress Hh ligands (Jiang and Hui, 2008). In addition to its oncogenic functions, our work suggests that Hh might also signal to local T-cells to decrease activation. Hh-driven suppression of a local T-cell immune response could uncover a previously unknown but targetable role for Hh signalling and/or Gli-dependent transcription in anti-tumour immunity.

## MATERIALS AND METHODS

### Mice

*Lck*-Gli2ΔN2 (Gli2A) (Rowbotham et al., 2007) and *Lck*-Gli2ΔC2 (Gli2R) (Rowbotham et al., 2008) C57BL/6-background transgenic mice were as described. All experiments were performed with littermate age-matched control mice under the ethical authority of the United Kingdom Home Office and local regulations.

### Cell culture

To activate T-cells, splenic cells at 5×10^6^ per ml were cultured in AIMV medium (Invitrogen) with 10^−5^ M β-mercaptoethanol (Sigma-Aldrich, UK) with or without soluble anti-CD3 and anti-CD28 (BD Pharmingen, UK, 0.01 μg/ml unless otherwise stated) or anti-CD3/CD28-coated beads (1:1 bead:cell ratio, Invitrogen Life Tech, UK), and with or without recombinant mouse IL-2 (Roche, UK, 10 U/ml) or Shh (R&D Systems, UK, 300 ng/ml). Magnetic bead separation was performed using EasySep CD4+ cell negative selection (StemCell Tech, France) according to the manufacturer's instructions.

### Flow cytometry, ELISA and antibodies

Cells were stained and analysed as described previously (Furmanski et al., 2013) on a BD C6 Accuri flow cytometer. CFSE labelling (10 µM in PBS) was performed as described (Rowbotham et al., 2007). Intracellular-cytokine and AnnV staining was performed using eBiosciences UK Fix/Perm and permeabilisation buffers and AnnV-binding buffers, respectively. Propidium iodide staining was performed in PBS immediately after cell surface staining, using 0.5–1.0 µg/ml propidium iodide (Sigma). Intracellular phospho-protein staining was performed using commercial buffers, BD Lyse/Fix and permeabilisation buffer III (BD Bioscience, UK), according to the manufacturer’s instructions. ELISAs were performed using eBioscience Ready-Set-Go mouse IL-2 kits (Affymetrix, UK). Data represent at least three experiments.

### Ca^2+^ flux

Splenocytes (1×10^6^–2×10^6^) were loaded with 3 µM Fluo-4-AM (Life Tech, UK) in Dulbecco's modified Eagle's medium (DMEM; Gibco, Life Tech, UK) for 30 min at 37°C, washed twice, surface-stained and washed. Cells for TCR stimulation were additionally surface stained with hamster anti-CD3 (3 µg per 10^6^ cells, BD, UK) and washed. All cells were resuspended in PBS containing Ca^2+^ and Mg^2^^+^ (Gibco, Life Tech, UK) and incubated at 37°C until acquisition. Data were acquired on a BD C6Accuri flow cytometer for 60 s prior to addition of stimuli (<9 µg anti-hamster IgG, Vector Labs UK; or 100 ng/ml Ionomycin, Sigma, UK).

### Microarray and data analysis

Array experiments were performed as described (Furmanski et al., 2013). Briefly, total RNA was extracted from purified fresh (unstimulated) or 6-h activated (stimulated) CD4+ spleen cells of WT (*n*=3), Gli2A (*n*=3) and Gli2R (*n*=3) and was submitted to UCL Genomics for processing and quality control prior to hybridisation to Affymetrix MOE430 2.0 mouse whole-genome array chips and data acquisition according to standard Affymetrix protocols. All microarray data are publicly available (dataset: GSE33156, GEO depository).

### qPCR

qPCR was carried out in triplicate as described previously (Furmanski et al., 2013). Pre-validated transcript-specific oligonucleotides (Quantitect Primer Assays, Qiagen, UK) were used except for Hoxa1F (5ʹ-CAGGGAAAGTTGGAGAGTACG-3ʹ) and Hoxa1R (5ʹ-TCTGCTTCATGCGGCGATTCTG-3ʹ). Data were normalised to expression of *Hprt* and represent at least two independent experiments.

### Western blotting

CD4+ cells from spleen (*n*=3 mice) were purified as described above and pooled for each group. Protein expression was analysed in nuclear or cell lysates by western blotting with appropriate antibody [against Gli1 (H-300), Gli2 (H-300) and IKKβ (H-470) (all Santa Cruz, UK); NFAT2 (D15F1, Cell Signaling Technology, UK) or control (anti-vinculin, Cell Signaling Technology; lamin A, Millipore; or actin, Santa Cruz Biotechnology)] as previously described (Bellavia et al., 2000; D'Acquisto et al., 2007). Briefly, cells were lysed in ice-cold lysis buffer (1% NP-40, 20 mM Tris-HCl pH 7.5, 150 mM NaCl, 1 mM MgCl_2_, 1 mM EGTA, 0.5 mM PMSF, 1 μM aprotinin, 1 μM leupeptin, 1 μM pepstatin, 50 mM NaF, 10 mM Na_4_P_2_O_7_, 1 mM NaVO_4_ and 1 mM β-glycerophosphate). Cell lysates were centrifuged at 18,000 ***g*** for 5 min at 4°C and the supernatants collected and subjected to electrophoresis on SDS 10% polyacrylamide gel. Membranes were incubated overnight with antibodies diluted in Tris-buffered saline solution containing Tween-20 with 5% non-fat dry milk at 4°C. In all cases, prior to western blotting, protein concentration in cell extracts was quantified using a colorimetric Protein Assay kit with reference to a BSA standard curve (Biorad, UK), according to the manufacturer's instructions. This information was used to load equivalent amounts of protein onto gels, which was confirmed using loading controls.

### EMSA and supershift experiments

LN cells of three mice per group and nuclear extracts probed by electro-mobility shift assays to analyse DNA binding of AP-1 or NFκB, as previously described (D'Acquisto et al., 2007). Briefly, nuclear extracts (3–5 μg) were incubated with 2 μg of poly (dI:dC) in 20 μl of binding buffer with ^32^P end-labelled, double-stranded oligonucleotide probes (5×10^5^ cpm), and fractionated on a 6% polyacrylamide gel (29:1 cross-linking ratio) in 0.5% TBE for 2.5 h at 150 volts. Double–stranded oligonucleotide probes were from Promega, UK. Supershift EMSA were performed as above, additionally using the following antibodies: anti-cFos, -FosB, -JunB, -JunD (all Cell Signaling Technology)

### Data analysis

Statistical analyses were performed using Microsoft Excel or Prism 4 (Graph Pad). Two-tailed unpaired Student's *t*-tests were used to assess statistical significance to *P*<0.05.
